# Blind Man’s Buff

**Published:** 1882-07

**Authors:** George E. Blackham


### BLIND MAN'S BUFF.

GEO. E. BLACKHAM, M. D.

This ancient and honorable game is well known, and in the main consists in inhibiting the use of vision and leaving the person so temporarily blinded to stumble about in the dark, 'till one of the other players is caught, and then the "blind man" is to *guess* who is caught. If the guess is correct, the person caught becomes in turn the blind man, and so the game goes on. If the guess is incorrect, the poor blind man must stumble about again, 'till he catches some one else, and *guesses* correctly. This is well enough for a "game," but it is not business.

No one would seriously propose that if you wished to accumulate knowledge, the best way would be to flounder about in the dark, 'till you stumbled accidentally over some fact, and then, keeping your eyes shut, to *guess* at what you have found.

And yet, practically, this is the system of education at the present day.

Children are not taught to *observe* and reason from their observations, but are given certain lessons to learn, and their standing in school is marked in direct ratio to their verbal memory. Thus the memory is trained at the expense of the observing and reflecting faculties.

Original observations and reflections are not encouraged, they are sedulously repressed. There is "no time" for them in the elaborate scheme of our graded schools.

We are still under the domain of the old scholastic idea in matters educational.

We hear much nonsense about the necessity of the study of the dead languages and mathematics, for mental training—learn so many Greek verbs, commit to memory so many lines of Virgil—be able to recite, parrot-like, the exact words of a demonstration of a problem or theorem of Euclid—tell correctly what the geography says about the name and location of the longest river in the world, or the highest mountain, and you will "pass" with honor.

It does not make the least difference whether you have any concrete idea of how long a mile is, or how high a thousand feet is—if you have correctly *memorised* what was written in your text-book you are all right.

The test of the excellence of any school seems to be the number of scholars who can "pass the Regents."

The aforesaid Regents being a series of questions propounded thrice a year by the Worshipful Regents of the University of the State of New York—and apparently prepared under the patronage of some hypothetical society for the diffusion of "useless information." The ability to "pass" depends almost absolutely upon the verbal memory of the scholars.

Of course by such a system a certain amount of information is in time accumulated, but the chief end and aim of school training is left almost entirely out of sight. This true aim of school life should be to fit scholars for actual life, to teach them how to ascertain the conditions of their environment and how to adapt themselves to, or to bend it to them.

A babe comes into this vale of tears as void of knowledge as it is bare of clothes—and learns more in the next four years than it does in double that period at any other time of life. It learns to walk, to speak a language, to see, to hear, to estimate distances, that fire is hot, and ice is cold, and a thousand other things which are useful to it, and necessary to its continued and healthful existence. Why does it learn so much in so short a time? Because its brain is more active, its intellect more receptive and its power of acquiring knowledge greater than in future life? Not at all. The more rapid acquirement is due to the class of facts presented and the method of education. What would be thought of the mother who instead of beginning to teach her child to speak, by saying, "This is mama—say mama. This is papa—say papa, dear." Should begin by teaching how many parts of speech there are and giving a list of regular and irregular verbs to study, with rules for their use. Yet this method is adopted for teaching foreign languages to children of older growth. What wonder, then, that "a little Latin and less Greek," is in a majority of cases the net outcome of the proceeding.

What if mama should—instead of holding baby up by the hands and encouraging and supporting its first faltering steps, teaching it to walk by the "experimental method"—lecture baby on the anatomy of its little legs, and the complex system of muscular co-ordination necessary to balance itself and step forward—baby might become a philosopher and know *why*, but he would be a very helpless *man*, and never know *how* to walk.

Baby is encouraged to "notice" things, to ask about them and to learn their properties in a purely practical way. He is not informed that "Heat is a mode of motion, which in excess may cause a solution of continuity or destruction of tissue, accompained by pain," but when he reaches for the lamp or candle he is told, "No—no—burn baby," but he don't *learn* this, though he may be able to *repeat* it, 'till he gets his little fingers against the hot lamp chimney or stove. *Then* he *learns*—he has made an experiment, he has learned to *observe*, and the knowledge thus acquired stays by him, and is of use to him all his life, and all the world knows that "A burnt child dreads the fire."

A boy who has been taught on the memorising plan quits school and goes to work in a drug store. He has *learned* apothecaries measure, and can sing-song his table:

> "Twenty grains make a scruple,
> Three scruples make one drachm,
> Eight drachms make one ounce."

He could "pass the Regents," in apothecaries measure, but he don't *know* anything about it, really. He don't *know* from actual observation what a fluid drachm or a fluid ounce, or a pint is. He can't tell a three ounce from a four ounce bottle. He has been playing at blind man's buff with apothecaries measure and he has finished his game and has to take the bandage off his eyes, and see and handle these things before he *knows* anything about them in a practical sense.

Now, that the bandage *is* off, he soon learns, he can soon tell a three ounce from a four ounce bottle. He learns to know how big a piece of paper he will need to do up a pound of salts. He learns all this, but not so quickly as he would learn it if he had been taught to use his observing faculties while he was in school—and besides this he ought to have been taught just these things in school and not have to learn them after he leaves it.

How many boys and girls leave school with a clear idea of an inch or a foot. Of course they have memorised the table that tells them that a foot is equal to twelve inches, and that an inch is the twelfth part of a foot.

But *how* long is an inch—how long is a foot? Do they know, do these terms convey any definite meaning to them? I think not as a rule, They have been playing at blind man's buff with the linear measure, and it is not till after the boy gets into the carpenter shop that he begins to know how thick an inch board is. He ought to have known it long before he left school.

See the dazed look of the average woman with a new sewing machine. She has to wait for the agent to show her how to take it apart, to set the needle and do every little detail, whereas if she had been trained in school to observe and reflect, to trace physical cause and effect, she could study the thing out for herself in short order.

It is related of Houdin, the great French magician, that he had so trained his observing faculties that in walking through a room once he could take a mental inventory of everything in it. Something like this children ought to be trained to do—but with this power ought to go the additional one of reflecting on what they see.

The world is so full of useful and wonderful things, that it seems strange that anyone could pass them by unnoticed—and yet the majority of people, thanks to faulty training, stumble about with hoodwinks on, playing blind man's buff with the phenomona of nature.

When a certain French priest, many years ago, wrote to his bishop, that by means of his telescope he had discovered spots on the face of the sun, the good prelate replied, in the spirit of scholasticism, "My dear son, I have looked through the works of Aristotle and find no mention of the spots you refer to; they must therefore be occasioned by imperfections in your eyes or your glasses. Think no more of them, but confine your attention to the writings of the fathers." The poor priest who had gotten his bandage off, put it on again and went on with his little game of "blind man's buff."

The boy in school who wants to know how big a foot is, is told to study his table and not ask foolish questions—a foot is twelve inches. "But how big is an inch?". "The twelfth part of a foot of course—don't interrupt the class with such silly questions."

"What is't ye read?" asks Polonious, "words, words, words," answers the crazy Prince of Denmark. What is't ye study? I ask of our common school scholars of to-day, and if they answered truly, they might use Hamlet's phrase, "Words, words, words." Names, not things; memory, not observation.

Even when the natural sciences are taught, the method is largely by means of text-books. Blind man's buff is the game, though the bandage is occasionally taken off, so that the scholar can see the professor (every pedagouge is a professor, save the mark) make an experiment or two.

The endeavor seems to be to make the scholar commit to memory what somebody has *said*, instead of encouraging him to find out for himself what they *really* are.

"Write me a good text-book on Natural History," said a great publisher to Louis Agassiz, "and name your price." "What we want is students not text-books," replied the great teacher. "The book of nature is always open, all we need to do is to open our eyes and look into it."

Suppose our school children were taught to observe, to note the coming and going of the birds and insects, the blossoming and fruiting of our native plants, that this one was useful for such and such reasons and that it was harmful for such and such reasons. Suppose their eyes were trained to estimate sizes and distances and their muscles to estimate weights; suppose they were trained to note every change in the ever shifting panorama of the world around them, to reason for themselves upon its causes and consequences; suppose, in fact, that the world had gotten old enough to stop playing blind man's buff, and that our children were encouraged to take off the bandage and taught to use the too long shaded eyes; suppose that in school they were taught things of use to them in their future struggle for existence, and trained in the art of seeing and learning for themselves.

Would'nt this be worth the sacrifice of the "little Latin and less Greek," of the knowledge of which mountain is *said* to be the highest in the world—in fact of all the sacrifices involved in the total removal from our educational system of the ancient and honorable, yet thoroughly absurd and useless game of blind man's buff.

